# Diversity and deadwood-based interaction networks of saproxylic beetles in remnants of riparian cloud forest

**DOI:** 10.1371/journal.pone.0214920

**Published:** 2019-04-12

**Authors:** Alfredo Ramírez-Hernández, Ana Paola Martínez-Falcón, Estefanía Micó, Sandra Almendarez, Pedro Reyes-Castillo, Federico Escobar

**Affiliations:** 1 CONACYT-IPICYT/Consorcio de Investigación, Innovación y Desarrollo para las Zonas Áridas, San Luis Potosí, México; 2 Centro de Investigaciones Biológicas, Universidad Autónoma del Estado de Hidalgo, Hidalgo, México; 3 Centro Iberoamericano de la Biodiversidad (CIBIO), Universidad de Alicante, Alicante, España; 4 Universidad Autónoma de San Luis Potosí, San Luis Potosí, México; 5 Instituto de Ecología, A. C. (INECOL), Xalapa, México; Universidade Federal de Mato Grosso do Sul, BRAZIL

## Abstract

We studied the saproxylic beetle community inhabiting deadwood in remnants of riparian cloud forests in “La Antigua” basin, in central Veracruz (Mexico). We assessed the influence of deadwood features (tree species, trunk position, trunk diameter, trunk volume and decomposition stages) on saproxylic beetle diversity. In order to assess the stability of beetle species-deadwood interactions, we also analyzed the ecological networks structure. A total of 63 deadwood trunks, belonging to four tree species, were sampled by standardized hand-collection throughout well-preserved remnants of riparian cloud forest. We found that tree species and deadwood decay stage are the main drivers that determine the diversity and stability of saproxylic beetle species interactions. Our results indicate that *Quercus corrugata* is the main tree species in terms of maintaining the significantly highest saproxylic beetle diversity, but with no stable interactions (saproxylic beetle-deadwood). A nested network structure was detected for *Clethra mexicana* and *Liquidambar styraciflua*, with a pool of core (generalist) saproxylic beetle species. We observed that beetle diversity from the early and late deadwood stages comprises distinct assemblages and the four stages of decomposition showed a nested network structure. During deadwood succession, community composition and guilds changed among networks; the early successional stage had more specialized xylophagous beetles, while other guilds (mycophagous, saprophagous and zoophagous) arrive later and become the core species in the advanced stages of decomposition networks. *Heliscus tropicus* (Passalidae) is a key species constituting the core of all of the networks and could be considered an ecosystem engineer in cloud forests. By exploring links between saproxylic beetles and deadwood characteristics, we can further our understanding of species interaction in order to develop management strategies oriented towards the protection of species and their habitats in this threatened ecosystem.

## Introduction

Deadwood has been widely studied over the last few decades [1,2] due to its contribution to maintaining functional biodiversity in forest environments [3,4–6]. Saproxylic (deadwood-dependent) species stand out as key organisms involved in the complex trophic webs of forests and other wooded environments, including some human modified habitats, such as crops with trees (e.g. shade coffee) and commercial plantations, among others [6–8]. During wood degradation, saproxylic beetles fulfilling important ecological functions [6] as a result of their capability to rapidly transform the physical-chemical properties of the microenvironment in which they develop their life cycle, directly influencing soil fertility and therefore also other saproxylic taxa [9–11].

Saproxylic beetle diversity has been utilized for conservation purposes since these species are highly sensitive to forest management and habitat degradation [6,12]. Furthermore, since deadwood is an ephemeral microhabitat, saproxylic beetles are highly vulnerable to wood extraction for fuel, among other human forest practices [12]. Studies in tropical forested environments have attempted to elucidate biological and ecological aspects of some beetle species of the families Scarabaeidae, Passalidae and Cerambycidae, which are directly associated with wood degradation [13–21]. Recently, Muñoz-López *et al*. [22] evaluated the whole saproxylic beetle community inhabiting deadwood from the tropical deciduous forests of central Mexico. Their study revealed that saproxylic beetle species are highly affected by both host tree species and wood decomposition stage.

Despite of an extensive body of research about diversity inhabiting deadwood, relatively few studies have focused on both diversity patterns and species interactions, the majority of the studies has been carried out on high latitude regions as European temperate and Mediterranean forests [23–25]. The patterns found on these latitudes and environments were summed up by Garrick et al., [26]. In this review they reported that the most common patterns structuring deadwood beetle communities are tree species, microhabitat conditions (e.g. log size), forest management and decomposition stages. However, little attention has been paid to tropical saproxylic beetle diversity [13] particularly from cloud forests, which is one of the most threatened ecosystems in the world [27].

In addition to studying the diversity patterns of saproxylic fauna, the comprehension of community structure under the framework of biotic interaction networks can help us to understand the ecological mechanisms related to the stability of interactions between species in order to gather evidence regarding the community resilience. Analysis of ecological networks is a useful tool that offers a wide number of emergent properties to give an overview of the way species interact and provides us with information concerning to their structure, function and dynamics [28–29]. Recent studies show that biotic interaction networks tend to be highly structured, and that some structural attributes not only promote the coexistence of species [28], but may also facilitate resilience and stability of the ecological systems in the face of disturbance [30]. Moreover, networks allow us to understand specialization throughout communities and the stability of interactions between species [28,31–33].

Saproxylic beetle species and their interactions vary to a broad extent, mainly due to factors associated with the host tree species, deadwood properties such as the deadwood position (standing or fallen), wood decomposition stage and microclimatic conditions, among others [2,34–36]. These variations influence the survival, reproduction and population persistence of saproxylic beetle species [2,6,37,38]. Saproxylic beetles exploit a spatio-temporally unpredictable resource (deadwood), and its availability may act to shape the saproxylic communities [39,40]. Deadwood is a key resource not only for saproxylic insects, but also communities of fungi as well as the predatory beetles that feed on other wood-inhabiting organisms. This interplay establishes a multitrophic food web within a deadwood tree [40].

In contrast with the wide research about the patterns of diversity inhabiting deadwood on high latitude forests, the deadwood-base interactions of saproxylic beetles are still insufficiently quantified also in those regions. The only few previous deadwood studies shows that decomposition stage and woodland complexity [23] may influence diversity and nestedness for saproxylic beetles [23,24–39,40]. In early stages, xylophagous beetles create suitable conditions for colonizing fungi and facilitate other species. Subsequently, saproxylic species access the nitrogen-fixing bacteria, so the communities interacting at different stages of the decomposition process are different and establish a great variety of trophic interactions [40]. Moreover, deadwood features such as size or chemical composition of the tree may regulate the communities inhabiting deadwood trees [24,25]. Tree species may create a saproxylic specialist community, feeding on a specific tree species, and deadwood decay stages may influence this specialization, because they facilitate the species that arrive at the beginning of the decomposition processes, while less specialized species can feed on the wood at more advanced stages of decomposition [40]. In this context, we aimed to determine whether patterns of deadwood-based interaction networks of saproxylic beetles were driven by niche-based matching of resource traits (e.g. deadwood decomposition stage) as in other latitude forests.

Specifically, this study explored the saproxylic beetle species inhabiting deadwood of the montane cloud forest, which occupies an area less than 1% of its original distribution and is consequently a threatened ecosystem in Mexico [41]. There are many factors that jeopardize cloud forests in Mexico, particularly in the State of Veracruz, that are mainly related to the unsustainable expansion of the agricultural frontier [42]. Accordingly, montane cloud forest in many of these human-dominated Mexican landscapes is restricted to the margin of rivers as riparian remnants of high value as biodiversity reservoirs, contributing to the maintenance of key important ecological functions [43–49] such as the natural flow of recycled nutrients during the deadwood decomposition process, which has been little studied to date. We therefore aimed to study the effect of deadwood features (tree species, position, diameter and decay stage) on saproxylic beetle diversity and the interaction networks between the beetles and deadwood in remnants of riparian cloud forest in central Veracruz (eastern Mexico). We addressed the following objectives: 1) to detect the main deadwood features that influence the richness and abundance of saproxylic beetles, 2) to compare the diversity of saproxylic beetles among the different deadwood features, and 3) to analyze the network structure between deadwood features and saproxylic beetle associations in order to disentangling beetle specialization patterns by tree species characteristics.

We hypothesize that tree species and deadwood decay stages should be key to determine both the saproxylic beetle species diversity and network structure. Therefore, we expected a shift of the higher values of diversity from the first stage of decomposition towards intermediate stages as initial stages are expected to be very short in time due of the high humidity and temperature in riparian cloud forests [50]. However, the higher species richness should not necessarily imply a greater stability in the interaction network. We establish that specialization is related to tree species and it should decrease with successional age of the deadwood. Furthermore, xylophagous species would be more abundant and interact with more trees; meanwhile other guilds (mycophagous, saprophagous and zoophagous) would arrive later when the wood has become more decayed. Thus, we would find nested interaction networks among tree species and decomposed stages giving by changes on specialization patterns on the different successional stages.

## Materials and methods

### Ethics statement

Prior to developing our study, permission to access privately owned land was obtained from all landowners. Permit No. 142-FAUT 0018 was provided to FE by the Dirección General de Vida Silvestre, Secretaría de Medio Ambiente y Recursos Naturales (SEMARNAT) for field scientific collections. No protected species were collected.

### Study area

Fieldwork was carried out in remnants of riparian cloud forest in the “La Antigua” basin, in central Veracruz. The mean annual temperature at the study area is between 12 and 18°C and precipitation ranges from 1,350 to 2,000 mm [51]. There are three pronounced seasons: a relatively dry and cool season from October to March, a dry and warm season between April and May, and a wet and warm season from June to September [42].

In general, the dominant vegetation in cloud forests varies in structure and composition because of the considerable topographic and environmental variation [46], which allows the coexistence of Temperate and Neotropical flora [42]. Some of the most representative tree species found in the cloud forests are *Liquidambar styraciflua var*. *mexicana* Oerst., *Quercus xalapensis* Humb. et Bonpl and *Q*. *leiophylla* A. D.C., among others [42].

### Sampling design and species identification

**Fig 1:**
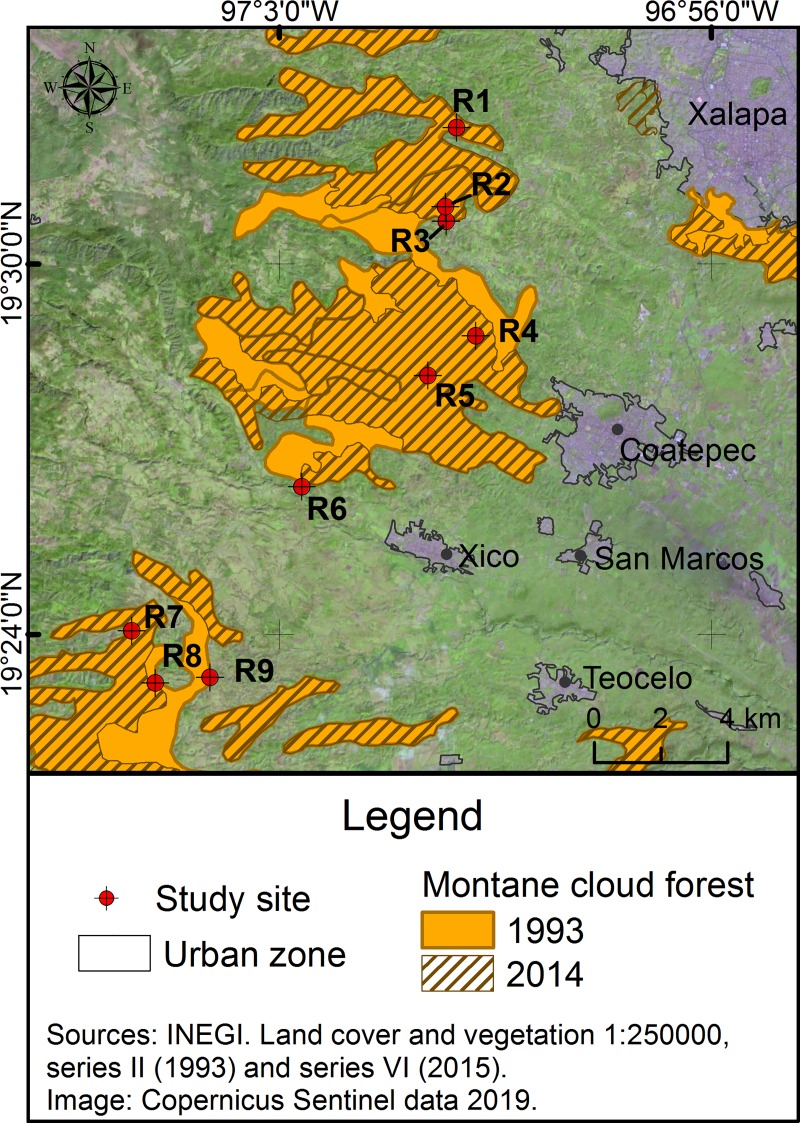
Location of the nine riparian remnants studied. Distribution of the nine riparian remnants of cloud forest throughout “La Antigua” Basin, in Central Veracruz (Mexico). In addition, the map also shows the change in cloud forest coverage between 1993 and 2004 in the basin.

A total of nine riparian vegetation remnants (Fig 1, S1 Table) were chosen based on the prior granting of access by the owners. Riparian remnants were separated by a distance ranging from 1 to 18 km. Patch size of the nine riparian remnants ranged from 1.5 to 9.7 ha, and these were located within an elevation range of 1,300 to 1,800 m.a.s.l. The riparian remnants studied were immersed in a mosaic of cloud forest patches, livestock farming, coffee plantations and human settlements, for which the extraction of firewood from the remnant forest is a very common practice [15,42,52].

Nine tree species typical of the cloud forest were identified (see S2 Table for more details) in the nine riparian remnants studied: *Alnus acuminata* Kunth 1817, *Clethra mexicana* DC., *Heliocarpus americanus* L., *Liquidambar styraciflua*, *Quercus corrugata* Hook, *Q*. *glabrescens* Benth., *Q*. *oleoides* Schltdl. & Cham., *Tabebuia rosea* (Bertol.) DC. (1845) and *Trema micrantha* (L.) Blume (1856). In each riparian remnant, we delimited a spike transect of 100 m in which nine deadwood trunks were searched intensively. A total of 81 deadwood pieces (57 logs and 24 stumps) were sampled; however, due to the very low number of logs and stumps with few saproxylic beetle species (<1 species) and individuals (<2 individuals) found (S2 Table), only the following four tree species were considered in this study for the analysis: *C*. *mexicana*, *L*. *styraciflua*, *Q*. *corrugata*, *T*. *micrantha*. A list of the nine tree species studied and the saproxylic beetle species collected at each is provided in S4 Table.

The sampling period was conducted between the end of the wet-warm season and the start of the dry-cool season (September and December 2015) because this represents the end of the main insect flying period and mating will already have taken place. There is therefore a likelihood of finding larva or teneral saproxylic beetles living inside the deadwood trunks to complete their life cycle. We used the dissection method for an initial rapid diversity assessment since it is considered crucial for a better understanding of species biology, habitat requirements and diversity distribution across habitats [53,54]; and is currently used in pioneering saproxylic studies [22,39]. Deadwood pieces were dissected by using a personal hunter ax and each one was sampled for one hour (total sampling of 81 hours). Saproxylic beetle specimens were collected manually using forceps and then placed into a container with 70% ethyl alcohol. Due to the lack of taxonomic references, the larvae were not sampled. Beetle species were identified by comparisons with the Entomological Collection of the Institute of Ecology (IEXA), and several Mexican specialists in Coleopteran taxonomy corroborated the species identification (see Acknowledgments). Due to the lack of taxonomic knowledge, some specimens were considered as morphospecies following the specialist corroboration. Since we do not have enough information on the trophic guild of the species, it is not included for formal analysis. A reference collection was deposited at the IEXA.

### Deadwood features

In order to characterize the deadwood, we measured the following variables:

1. Tree species (categorical variable).
2. Position (categorical variable), defined by fallen (logs) or standing (stumps) trunks.
3. Tree length (continuous variable). Logs and stumps ˃30cm of length were considered in this study.
4. Deadwood average diameter (continuous variable). Measured at three points for logs (top, middle and base), whereas stumps were measured by taking into account the diameter in the middle. Deadwood diameters of each trunk were averaged and ranged from 20 to 140 cm (S3 Table).
5. Diameter classes: With regard to different ranges of average diameter, we determined three diameter classes as follows: C1 <50 cm; C2, 50–100 cm; C3 100–150 cm (S3 Table).
6. Deadwood volume: The volume (cm^3^) of the deadwood pieces was estimated using Newton's Truncated Cone Volume with the following formula:
  $$Vtc=\frac{L}{3}(S_{0}+S_{1}+\sqrt{S_{0}S_{1}})$$
  where *Vtc* is the total truncated cone volume of deadwood trunks, *S*_*0*_ is the greater section area at the base of the trunk, *S*_*1*_ is the smaller section area at the apex of the trunk and *L* is the length (or height) of both logs or stumps [55].
7. Deadwood stages: A penknife was used, and field observations were undertaken. Four deadwood decomposition categories (S3 Table) were determined following Franc et al. [56] and some personal observations during fieldwork: D-I, wood was hard and presented resistance to penetration with the knife, bark with moss firmly attached to the stem; D-II, soft bark partly loose but the inner wood was still hard to penetrate with the knife; D-III, soft and wet wood and the inner wood was still hard to penetrate with an increase of moss and fungus presence, the knife was able to penetrate the wood (1–5 cm); D-IV, bark loose and mostly gone and the knife penetrated the wood with no resistance for more than 5 cm and the wood is easily broken by hand or exhibits a high level of decomposition and water accumulation with fungus. Higher soil moisture was commonly present.

### Data analysis

#### Deadwood characteristics

Generalized Linear Models (GLM) were used to explore differences in saproxylic beetle species richness and abundance among all deadwood features. Since the data were counts, we constructed models using Poisson error distribution and log-link function and then checked for over-dispersion [57]. The lowest value of the Akaike Information Criterion (AIC) was also used to select the most probable model. These analyses were carried out using R 3.2.1 software [58].

#### Diversity patterns

Sample coverage (Ĉ*n*) was used to evaluate inventory completeness for deadwood features (i.e., tree species and decomposition stage) [59]. Values of Ĉ*n* range from 0 (minimal completeness) to 100% (maximum completeness), and it is a robust and commonly used measure of sampling completeness [60].

In order to detect differences in saproxylic beetle diversity between deadwood features, the Shannon diversity (^1^*D*) was calculated. This diversity is part of the Hill numbers [61,62] and indicates the number of typical or common species within the community. Shannon diversity does not favor rare or dominant species, since all species are weighted according to their relative frequency in the sample. For this reason, it is considered a more informative measure of diversity than species richness (^0^*D*) or Simpson diversity (^2^*D*), see details in Jost [62]. For comparisons of Shannon diversity, the 95% CI was used and differences were determined following the recommendations of Cumming et al. [63], where an absence of overlap between CI values indicates a significant difference. Given the presence of rare species (singletons) in the riparian cloud forests, we calculated the estimated diversity by employing the Chao and Shen method, which produces an accurate estimation when there are unrecorded species in a community [64,65]. The sample coverage estimator and diversity of order ^1^*D* were calculated using the SPADE software [65].

Differences in species composition were estimated by calculating the Jaccard Similarity Index [66,67], which is based on the relationship between the number of species shared between two sample units and the total number of species [68]. An analysis of similarity (ANOSIM) [69] was performed to test the significance of these differences using PRIMER software v6 [70].

#### Network metrics

We built individual-based networks using saproxylic beetles associated with each deadwood features. We used the *NODF*-metric [71] in the software ANINHADO to measure the degree of nestedness for each network [72]. We tested nestedness using Null Model II [31]. For quantitative networks, we used *WNODF* (Weight Nestedness Metric Based on Overlap and Decreasing Fill) and tested the *WNODF* significance using the Null Model RC [71]. Both nestedness types vary from zero (no nestedness) to 100 (perfect nestedness) [33]. We also estimated the modularity for each network using the Modularity Index M (from 0: no subgroups, to 1: completely separated subgroups), based on Newman’s algorithm obtained through simulating annealing (1000 randomizations performed), using the Modular software [73].

We used the package “bipartite” [74] of R software [58] to plot network graphs and calculate the following metrics [75]: (i) links per species (sum of interactions divided by the number of saproxylic beetles); (ii) connectance (the proportion of links realized from the total possible links in each network, defined as the sum of links divided by the number of cells in the matrix); (iii) *H*_2_ specialization index, ranging from 0 (lower specialization) to 1 (high specialization) [32]. Network graphs were constructed using R software [58].

The categorical core versus periphery analysis was used to describe species that constituted the core (generalist species: those with the most interactions) or peripheral (those with fewer interactions) components of the network (*n* = 20 randomizations/network). This procedure helps to evaluate the importance of each saproxylic beetle species within the network [76,77]. Core-periphery analysis was performed with UCINET for Windows 6.0 [78].

## Results

From 63 deadwood pieces (49 logs and 14 stumps) belonging to four tree species, a total of 387 individuals and 44 saproxylic beetle species (21 morphospecies), belonging to nine families (Carabidae, Dynastidae, Leiodidae, Passalidae, Ptilodactylidae, Scarabaeidae, Staphylinidae, Tenebrionidae and Zopheridae) of saproxylic beetles, were collected (S4 Table). The richest families were Staphylinidae (18 species) and Carabidae (9 species), while the most abundant were Passalidae (171 individuals) and Staphylinidae (156 individuals). With regard to the most abundant beetle species, we found *Heliscus tropicus* (Percheron, 1835) represented by 41% (Passalidae) of the individuals collected, followed by *Osorius* sp.1 with 17% (Staphylinidae). In addition, a total of 17 species (39%) were recorded at very low frequencies (S4 Table).

### Deadwood characteristics

GLM showed that saproxylic beetle abundance differed significantly for tree species (*χ*^2^ = 72.60, *df* = 3, *p*<0.001) and decomposition stage (*χ*^2^ = 22.92, *df* = 3, *p* <0.001), but not for position (*χ*^2^ = 2.10, *df* = 1, *p* = 0.14), length of pieces of deadwood (*χ*^2^ = 0.90, *df* = 1, *p* = 0.34), average diameter of deadwood (*χ*^2^ = 0.63, *df* = 3, *p* = 0.42) and volume of deadwood (*χ*^2^ = 1.96, *df* = 1 *p* = 0.16).

Other models were also constructed by creating categorical factors from the original field measurements of deadwood variables, according to tree species, position and average diameter, to describe diameter classes and decomposition stages. Again, GLM abundance results showed significant differences for tree species (*χ*^2^ = 72.60, *df* = 3, *p*<0.001) and decomposition stage (*χ*^2^ = 18.47, *df* = 3, *p* <0.001); but not for position (*χ*^2^ = 2.10, *df* = 1, *p* = 0.14) or diameter class (*χ*^2^ = 1.38, *df* = 2, *p* = 0.50).

Neither model using Poisson or Quasipoisson distribution was significant for saproxylic beetle species richness. We only used the significant deadwood categorical characteristics (tree species and deadwood decomposition stage) to explore differences in diversity patterns and network structure metrics.

### Diversity patterns

Sampled coverage ranged from 77 to 95% and diversity comparisons showed significant differences (at CI 95%) among tree species and decomposition stages (Table 1). With respect to tree species, *Q*. *corrugata* showed the highest diversity, which was twice that of *T*. *micrantha* (Table 1). We did not find differences between *C*. *mexicana* and *L*. *styraciflua*. In contrast, *Q*. *corrugata* was three times more diverse than *C*. *mexicana* and *L*. *styraciflua* (Table 1). On the other hand, the decomposition categories D-I, D-II and D-IV were almost two times more diverse than D-III (Table 1).

**Table 1:** Diversity and network patterns. Relationship of the diversity estimators and network values found through the four tree species and the four deadwood decomposition stages evaluated from the riparian cloud forest in the “La Antigua” basin, central Veracruz.

Diversity and network descriptors	Tree species				Decomposition stages			
	*C*. *mex*.	*L*. *sty*.	*Q*. *cor*.	*T*. *mic*.	D-I	D-II	D-III	D-IV
No. of deadwood pieces	19	16	19	9	20	27	7	9
Beetle richness	21	16	23	12	20	30	7	14
No. of individuals	145	149	62	31	100	215	29	43
Ĉ*n* (%)	94	95	84	77	91	93	93	83
^1^*D±*IC95%	7.9±1.1	6.7±0.9	21.7±3.1	11.5±2.9	12.3±1.9	9.6±1.4	5.6±1.1	10.6±2.3
Qualitative Nestedness (NODF metric)	13.89^*ns*^	20.28^*ns*^	9.30^*ns*^	13.24^ns^	12.08^*ns*^	10.59^*ns*^	37^*ns*^	22.97^*ns*^
Quantitative Nestedness *(WNODF)*	7.09*	6.46*	3.77^*ns*^	4.90^*ns*^	7.43*	5.90*	6.00*	5.87*
Modularity	0.58^*ns*^	0.47^*ns*^	0.25^*ns*^	0.66^*ns*^	0.21^*ns*^	0.62^*ns*^	0.47^*ns*^	0.55^*ns*^
Links per species	1.17	1.12	0.97	0.85	1.02	1.1	1	1.04
Connectance	0.11	0.14	0.09	0.16	0.1	0.07	0.28	0.19
*H*_*2*_	0.57	0.59	0.44	0.59	0.59	0.63	0.46	0.65

C. mex.: Clethra mexicana, L. sty.: L. Liquidambar styraciflua, Q. cor.: Quercus corrugata, T. mic.: Trema micrantha.

D-I: Hard wood with presence of moss and vegetation; D-II: Hard wood inside with soft bark and presence of moss and fungi; D-III: Soft and moist wood, hard at the center with a high increase of moss and fungi; D-IV: Soft wood, very humid and decomposed with the presence of fungi at the base of the trunk and on the ground

Ĉ*n*: Sample coverage estimator

^1^*D*: Inverse of the Shannon diversity index

* indicates differences at *p* <0.001 and *ns* indicates no significance.

Jaccard similarity index values ranged between 22 to 32% of species shared for tree species and 10 to 32% of species shared for decomposition stage. However, with regard to the analysis of similarity (ANOSIM) there were no significant evidence of species turnover among tree species (R = 0.02, *p* = 0.17) or decomposition stages (R = -0.01, *p* = 0.60).

### Deadwood-based interaction networks

**Fig 2:**
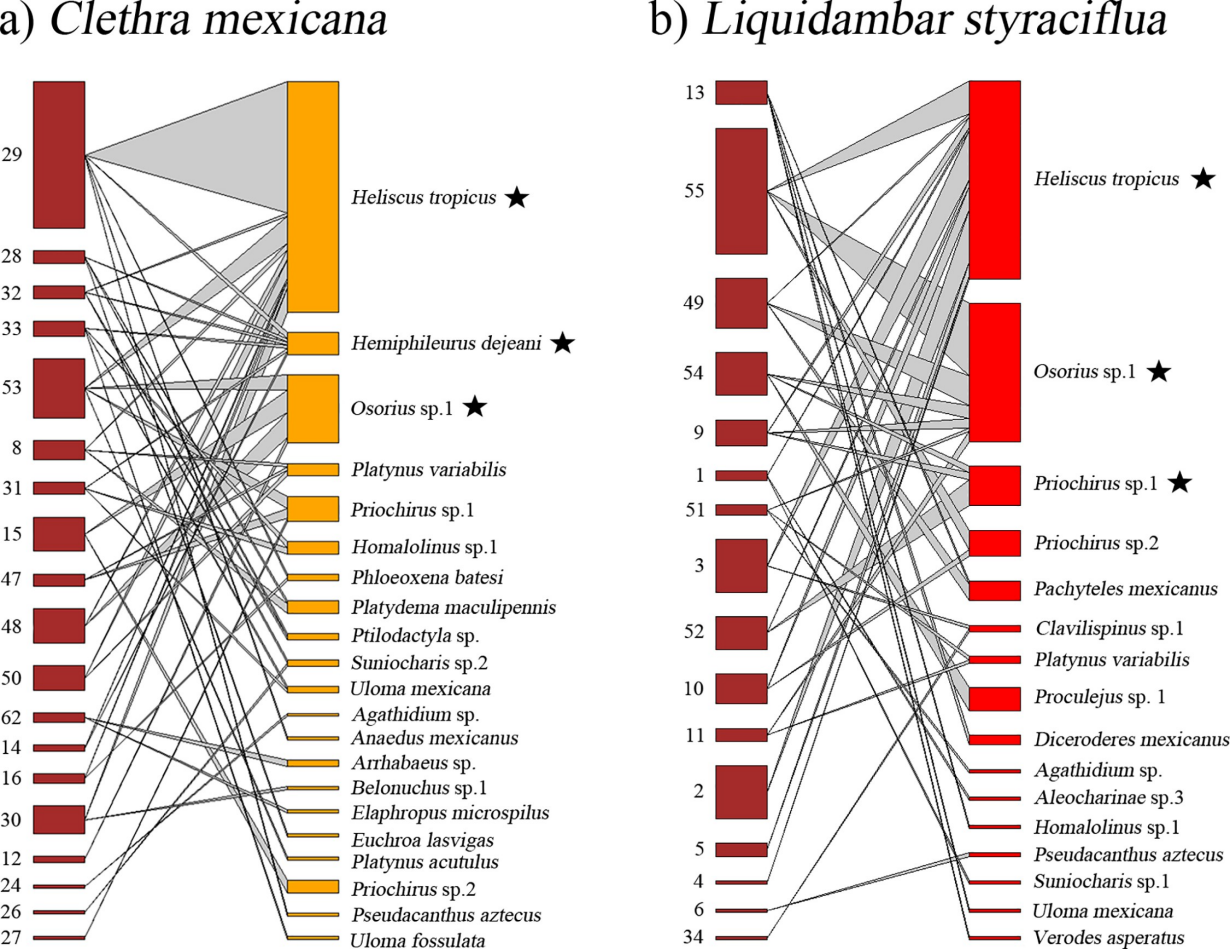
Saproxylic beetle network between two tree species. Differences in core composition related to a) *Clethra mexicana* and b) *Liquidambar styraciflua* tree species. Nodes on the left correspond with each sampled trunk and nodes on the right represent the saproxylic beetle species. Species that constitute the core of the network are highlighted with a black star.

**Fig 3:**
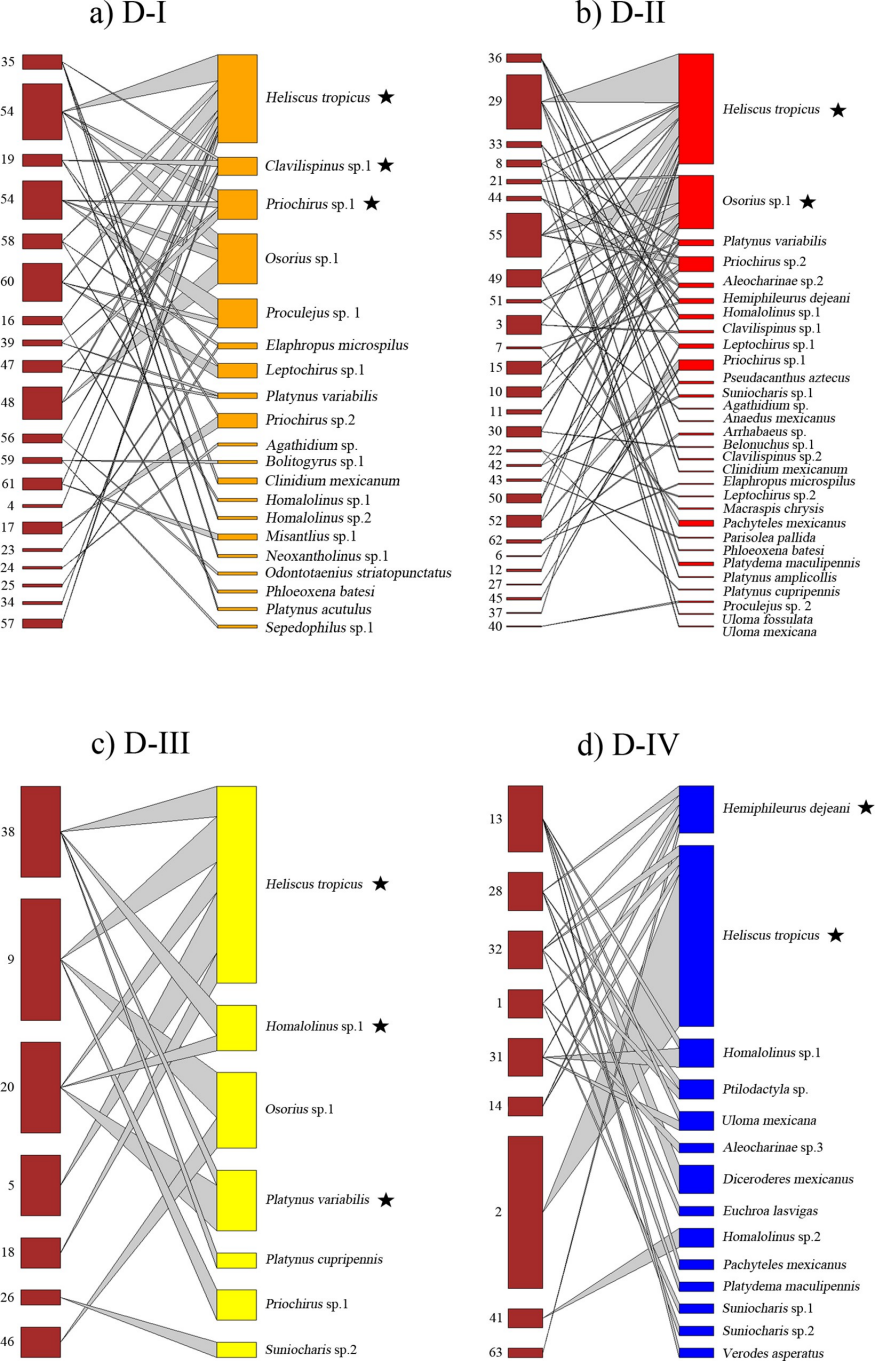
Saproxylic beetle network among decomposition stages. Differences in core composition related to each of deadwood decomposition stages, where D-I represents the initial stage, D-II, D-III the intermediate stages and D-IV the most advanced stage. Nodes on the left correspond to each trunk sampled and nodes on the right represent the saproxylic beetle species. Species that constitute the core of the network are highlighted with a black star.

Quantitative networks showed a significant-nested pattern of interactions for the tree species *C*. *mexicana* (*WNODF* = 7.09, *p* = 0.001) and *L*. *styraciflua* (*WNODF* = 6.46, *p* = 0.001) (Table 1, Fig 2) and for all decomposition stages (Table 1, Fig 3). Network attributes were similar among tree species and within decomposition stage networks (Table 1). In contrast, qualitative deadwood-based interaction networks did not exhibit a nested pattern of interactions for tree species and decomposition stage (Table 1). None of the studied tree species or decomposition stage-saproxylic beetle species networks presented a significant modular pattern (Table 1).

As regard to the categorical core *vs* periphery comparison of saproxylic beetle species networks, we found three species constituting the core of *C*. *mexicana* (Fig 2A) and also three species related to *L*. *styraciflua* (Fig 2B). With respect to the decomposition network, we found three species related to D-I category (Fig 3A), two to the D-II category (Fig 3B), three to the D-III category (Fig 3C) and two associated with the D-IV category (Fig 3D). In all cases, the main core species was the bess beetle *H*. *tropicus*, which was the most abundant beetle species (Figs 2 and 3).

## Discussion

As we hypothesized, our results provide evidence that host tree species and deadwood decomposition stage are the main drivers of the saproxylic beetle diversity in tropical cloud forest in central Veracruz, Mexico. This is consistent with findings in saproxylic beetle communities inhabiting deadwood in forests from other latitudes [27]. Regarding patterns in ecological networks, our results coincides with Quinto et al [23] where tree species influence the network structure promoting a nested pattern. Saproxylic diversity was higher in *Q*. *corrugata*; however, this tree species does not support stable species interactions. The saproxylic network structure was thus significantly nested for both *C*. *mexicana* and *L*. *styraciflua*. Moreover, the four deadwood stages showed differences in species diversity and, in all cases, the networks were nested. In general, our findings agree with those of Muñoz-López *et al* [22], who reported a similar diversity pattern in a tropical deciduous forest of central Mexico.

It is recognized that tree species act as a keystone element for forest biodiversity [6,79]. In particular, the *Quercus* genus is known to be key for saproxylic diversity in Temperate and Mediterranean forest, maintaining a high number of threatened saproxylic beetle species [6,8,35,80]. Our results showed that *Q*. *corrugata* supports a saproxylic diversity that is three times greater than that found in the other three tree species (Table 1), as well as the highest species richness. This tree species belongs to the group of white oaks, which are characterized by the hardness of their wood [81]. Consequently, their natural degradation after death could take longer than five years [82]. This long-term degradation process would constitute a more stable microhabitat for species with narrow host specificity, as is the case of the saproxylic beetles associated with the earlier stages of decay [6]. Nevertheless, the conservation status of this tree species could endanger the diversity with which it is associated. According to González-Espinosa et al. [83], this tree species is severely affected by unsustainable practices and should be categorized as Endangered in the red lists. Conservation of this species is therefore crucial for the maintenance of forest biodiversity.

Our results also reveal that saproxylic beetle s found in early and late deadwood stages comprise species with distinct trophic guilds, a pattern that has previously observed in Tropical forests [16,22]. The high diversity recorded in D-I was dominated by pioneer beetle species. However, as decomposition advances, other beetle species colonize the decayed trunk, which then establishes a more complex community with zoophagous, mycophagous and saprophagous beetles interacting among themselves, producing increased species diversity at the most advanced stages of decomposition [16,22] (Table 1). According to our prediction, there is a dominance of xylophagous species in early decomposition stages, e.g. the bess beetle *H*. *tropicus*, a core species in all networks but mainly in D-I with more interaction frequency and abundance in individual trunks. With progress through the deadwood stages, *H*. *tropicus* individuals interact with diminishing frequency in individual trunks and congregate in only a few, although they never disappear entirely. Early successional saproxylic beetles act as initiators of deadwood decomposition by facilitating the entrance of secondary colonizers.

Bess beetles were the most abundant because these species exhibit a sub-social behavior where the parents care for and feed their offspring. They feed on the wood and create galleries, through which they facilitate the entry of other insects, fungi and bacteria that contribute to the decomposition of the trunk [84]. Thus, their presence shapes the microenvironmental conditions that facilitate colonization by a wide number of invertebrate species and other associated taxa [18,19]. This suggests that bess beetles and particularly *H*. *tropicus*, can be considered ecosystem engineers in tropical cloud forests.

We found that D-II had the greatest network size, with more species interacting and where other beetle species of sapromycophagous habits (*Osorius* sp., family Staphylinidae) become core species. Our data reveal an unexpected low interaction frequency in the late successional stages, in contrast to Fierro et al. [39] who found higher colonization rates at late successional stages due to more suitable habitat conditions and an increased abundance of reproductive adults. It is likely that other arthropods increase in abundance, increasing the abundance of predator beetles and thus affecting the network size for beetles in the more advanced decomposition states.

Generally, with an increase in the number of interactions, it is expected that the nested structure of community might be reinforced, since nestedness is strongly associated with the connectivity of the network [85]. It is recognized that nested ecological networks are more resilient to species loss [86,87]. The nested structure of the saproxylic beetle networks from tree species in cloud forests are probably mainly influenced by abiotic factors, such as high levels of humidity, which cause wood degradation to occur faster than in Temperate forests [88]. Thus, the renewal of the community is constant and network attributes, as well as nested structure, are the result of the stability of the saproxylic community. In addition, we found a set of species forming the (generalist) core of the networks that shift between tree species as well as shifting between wood decay stages, in contrast to the few peripheral (specialist) species.

Our results also reveal that the saproxylic beetle network presents stability and low-connectance, with values ranging from 0.09 to 0.16 for tree species and from 0.07 to 0.028 for the four deadwood decomposition stages (Table 1). Studies conducted by Quinto *et al*. [23,89] have also exhibited low connectance values with regard to saproxylic beetles inhabiting tree hollows (0.099) and in three Mediterranean woodlands (0.11–0.15) of central Spain. The results highlight the fact that saproxylic beetle networks exhibit low connectance values that show highly generalist beetle species. Low connectance gives high stability to the network interactions since it reduces the negative impact of species extinctions throughout the network, suggesting greater resilience [86] and favoring the dynamics of wood degradation in the Tropical cloud forest. Other important aspect to consider, was that the later decomposition networks were smaller that initial ones (D-I and D-II had the double network size than the D-II and D-IV, Table 1), which may limit the power of the analysis, and strong conclusions could also be limited. Notwithstanding these results reflects the successional natural species replacement of this deadwood-beetle network system in tropical montane cloud forest.

In conclusion, this is the first attempt to understand and compare deadwood ecology (diversity patterns and beetle network interactions) in a tropical cloud forest. Our study showed that tree species and decomposition stages determinate saproxylic beetle diversity in this threatened ecosystem. These results are in agreement with other studies that consider this vegetation type a hotspot for saproxylic species [5]. Owing to the restricted distribution of remnant riparian cloud forests, the availability of deadwood could be limited. Thus, special attention must be given to evaluation of the effect of human activities such as the extraction of deadwood for fuel, which could act as a driving force by negatively affecting the saproxylic diversity [12]. Further studies on saproxylic beetle diversity are required in order to understand the way that species respond to habitat modifications or other factors such as the chemical characteristics of each host tree species over the course of the deadwood degradation process [40,89]. A long-term study is necessary in order to understand the entire wood decomposition process and to acquire knowledge regarding the spatiotemporal successional patterns that influence saproxylic diversity and fungi colonization due to saproxylic beetles. In addition, we suggest sustainable activities that maintain deadwood pieces on the forest floor, with only a moderate extraction of wood for fuel. This might contribute to the conservation of the associated species and act to improve the structure and the natural balance of processes in cloud forests. By exploring the links between saproxylic beetles and other deadwood characteristics, we can utilize a well-understood species interaction to develop management strategies oriented towards the protection of species and their habitats in this threatened ecosystem.

## Supporting information

S1 Table Geographical coordinates. Names and coordinates of the nine remnants of riparian cloud forest in the “La Antigua” basin of central Veracruz, Mexico.
(DOCX)

S2 Table Tree species and distribution in Veracruz State. List of the nine tree species studied in remnants riparian with their common names and their distribution among Veracruz State.
(DOCX)

S3 Table Tree species features. List of the nine typical tree species from Cloud Forest with the characteristics found among deadwood pieces from each tree species sampled.
(DOCX)

S4 Table Checklist of the saproxylic beetle species. Saproxylic beetle species and abundance found at each tree species from remnants riparian cloud forest, in “La Antigua” basin; central Veracruz.
(DOCX)
