# Supplementary material for: Diversity and deadwood-based interaction networks of saproxylic beetles in remnants of riparian cloud forest
Source: PLoS One. 2019 Apr 12;14(4):e0214920. doi: 10.1371/journal.pone.0214920 (PMC6461242; doi:10.1371/journal.pone.0214920)
Supplement: S1 Table — Names and coordinates of the nine remnants of riparian cloud forest in the “La Antigua” basin of central Veracruz, Mexico. (DOCX) [file pone.0214920.s001.docx]

**Supporting information**

**S1 Table. Geographical coordinates.** Names and coordinates of the nine remnants of riparian cloud forest in the “La Antigua” basin of central Veracruz, Mexico

| **Remnants of Riparian Cloud Forest** | **Geographical Coordinates** | | **Altitude**  **(m a.s.l)** |
| --- | --- | --- | --- |
|  | **Longitude** | **Latitude** |  |
| Pixquiac (R1) | 97° 0'08.47"W | 19°32'14.57"N | 1568 |
| Capulines (R2) | 97° 0'17.06"W | 19°30'56.40"N | 1619 |
| Arboleda (R3) | 97° 0'18.53"W | 19°30'41.30"N | 1625 |
| Riscal (R4) | 96°59'48.77"W | 19°28'51.15"N | 1551 |
| La Granada (R5) | 97° 0'36.10"W | 19°28'11.26"N | 1440 |
| Matlalapa (R6) | 97° 2'38.52"W | 19°26'24.89"N | 1627 |
| Vista Hermosa (R6) | 97° 5'22.56"W | 19°24'03.05"N | 1824 |
| Tlalchy (R7) | 97° 4'59.64"W | 19°23'13.50"N | 1644 |
| Puente de Dios (R8) | 97° 4'07.89"W | 19°23'17.16"N | 1538 |
