# Supplementary material for: Diversity and deadwood-based interaction networks of saproxylic beetles in remnants of riparian cloud forest
Source: PLoS One. 2019 Apr 12;14(4):e0214920. doi: 10.1371/journal.pone.0214920 (PMC6461242; doi:10.1371/journal.pone.0214920)
Supplement: S2 Table — List of the nine tree species studied in remnants riparian with their common names and their distribution among Veracruz State. (DOCX) [file pone.0214920.s002.docx]

**S2 Table. Tree species and distribution in Veracruz State.** List of the nine-tree species studied in remnants riparian with their common names and their distribution among Veracruz State.

| **Tree species** | **Common**  **name** | **Vegetation type** | **Status** | **Leaf type** | **Hardness**  **of wood** | **Uses** |
| --- | --- | --- | --- | --- | --- | --- |
| *Alnus acuminata* Kunth, 1817 | Litle | Cloud forest, Tropical forest | Native-Endemic | Evergreen | Soft | Wood for fuel |
| *Clethra mexicana* DC. | Marangola | Cloud forest, Oak forest | Native-Endemic | Evergreen | Soft | Wood for fuel |
| *Heliocarpus americanus* L. | Jonote | Cloud forest | Native | Deciduous | Soft | Wood for fuel, timber-yielding |
| *Liquidambar styraciflua* L. | Liquidambar, Ocozote | Cloud forest, Tropical forest | Native | Evergreen | Soft | Craftwork, timber-yielding |
| *Quercus corrugata* Hook. | Encino | Cloud forest | Native | Evergreen | Hard | Wood for fuel |
| *Quercus glabrescens* Benth. | Encino | Cloud forest | Native | Evergreen | Hard | Wood for fuel |
| *Quercus oloides* Schtltdl & Cham | Duela | Cloud forest | Native | Evergreen | Hard | Wood for fuel |
| *Tabebuia rosea* (Bertol) DC. 1845 | Rosa morada | Cloud forest | Wild tree | Deciduous | Soft | Timber-yielding |
| *Trema micrantha* (L.) Blume, 1856 | Ixpepe | Cloud forest | Wild tree | Evergreen | Soft | Wood for fuel |
