# Supplementary material for: Diversity and deadwood-based interaction networks of saproxylic beetles in remnants of riparian cloud forest
Source: PLoS One. 2019 Apr 12;14(4):e0214920. doi: 10.1371/journal.pone.0214920 (PMC6461242; doi:10.1371/journal.pone.0214920)
Supplement: S3 Table — List of the nine typical tree species from Cloud Forest with the characteristics found among deadwood pieces from each tree species sampled. (DOCX) [file pone.0214920.s003.docx]

**S3 Table. Tree species features.** List of the nine typical tree species from Cloud Forest with the characteristics found among deadwood pieces from each tree species sampled

| **Tree species** | **Position** | **Average Diameter (cm)** | **Diameter Range (min. – max.)** | **Decomposition stages** |
| --- | --- | --- | --- | --- |
| *Alnus acuminata* | Stump | (C2) 52.69 | 52.00 - 54.08 | D-I |
| *Clethra mexicana* | Log | (C1) 36.73 | 20.33 - 48.66 | D-II, D-IV |
|  |  | (C2) 58.60 | 50.33 - 72.24 | D-I, D-II, D-IV |
|  |  | (C3) 106.33 | 104.33 - 109.33 | D-I |
|  | Stump | (C1) 38.33 | 38.33 - 38.33 | D-I |
|  |  | (C2) 59.51 | 54.30 - 60.80 | D-I, D-II |
|  |  | (C3) 100.11 | 100.11 - 100.11 | D-III |
| *Heliocarpus americanus* | Log | (C1) 26.00 | 26.00 - 26.00 | D-I |
| *Liquidambar styraciflua* | Log | (C1) 34.13 | 21.33 - 46.96 | D-I, D-II, D-III, D-IV |
|  |  | (C2) 70.35 | 65.74 - 76.07 | D-I, D-II, D-III |
|  |  | (C3) 107.76 | 107.76 - 107.76 | D-II |
|  |  | (C4) 195.77 | 195.76 - 195.76 | D-II |
|  | Stump | (C1) 44.00 | 44.00 - 44.00 | D-IV |
|  |  | (C2) 83.93 | 71.66 - 87.11 | D-IV |
|  |  | (C3) 122.10 | 103.31 - 140.88 | D-I, D-II |
|  |  | (C4) 163.06 | 163.06 - 163.06 | D-I |
| *Quercus corrugata* | Log | (C1) 31.92 | 22.66 - 44.33 | D-I, D-II, D-III, D-IV |
|  |  | (C2) 67.04 | 51.96 - 99.92 | D-III, D-IV |
|  |  | (C3) 116.71 | 116.71 - 116.71 | D-IV |
|  | Stump | (C2) 64.58 | 57.66 - 85.33 | D-II |
|  |  | (C3) 119.91 | 119.91 - 119.91 | D-I |
| *Quercus glabrescens* | Stump | (C4) 183.00 | 183.00 - 183.00 | D-II |
| *Quercus oleoides* | Log | (C1) 46.07 | 46.07 - 46.07 | D-IV |
|  | Stump | (C1) 49.00 | 49.00 - 49.00 | D-II |
|  |  | (C2) 85.00 | 85.00 - 85.00 | D-II |
|  |  | (C4) 185.97 | 185.97 - 185.97 | D-II |
| *Tabebuia rosea* | Stump | (C2) 64.00 | 64.00 - 64.00 | D-I |
| *Trema micrantha* | Log | (C2) 80.33 | 65.66 - 92.66 | D-I |
|  |  | (C3) 112.00 | 112.00 - 112.00 | D-I |
|  | Stump | (C1) 47.00 | 47.00 - 47.00 | D-I |
|  |  | (C2) 89.83 | 64.33 - 99.33 | D-I, D-III |
|  |  | (C3) 138.43 | 138.43 - 138.43 | D-II |

**C1**: 0-50cm; **C2**: 50-100 cm; **C3**: 100-150 cm; **C4**: 150-200 cm.

**D-I**: Hard wood with presence of moss and vegetation; **D-II**: Hard wood inside with soft bark and presence of moss and fungi; **D-III**: Soft and moist wood, hard at the center with a high increase of moss and fungi; **D-IV**: Soft wood, very humid and decomposed with the presence of fungi at the base of the trunk and on the ground
