# Supplementary material for: Diversity and deadwood-based interaction networks of saproxylic beetles in remnants of riparian cloud forest
Source: PLoS One. 2019 Apr 12;14(4):e0214920. doi: 10.1371/journal.pone.0214920 (PMC6461242; doi:10.1371/journal.pone.0214920)
Supplement: S4 Table — Saproxylic beetle species and abundance found at each tree species from remnants riparian cloud forest, in “La Antigua” basin; central Veracruz. (DOCX) [file pone.0214920.s004.docx]

**S4 Table. Checklist of the saproxylic beetle species.** Saproxylic beetle species and abundance at each tree species from remnants riparian cloud forest, in “La Antigua” basin; central Veracruz

| **Families** | **Species** | **Tree species** | | | | | | | | |
| --- | --- | --- | --- | --- | --- | --- | --- | --- | --- | --- |
|  |  | ***Alnus***  ***acuminata*** | ***Clethra***  ***mexicana*** | ***Heliocarpus***  ***americanus*** | ***Liquidambar***  ***styraciflua*** | ***Quercus***  ***corrugata*** | ***Quercus***  ***glabrescens*** | ***Quercus***  ***oleoides*** | ***Tabebuia***  ***rosea*** | ***Trema***  ***micrantha*** |
| **Carabidae** | *Clinidium mexicanum* | 0 | 0 | 0 | 0 | 3 | 0 | 0 | 0 | 0 |
|  | *Elaphropus microspilus* | 0 | 1 | 0 | 0 | 1 | 0 | 0 | 0 | 1 |
|  | *Euchroa lasvigas* | 0 | 1 | 0 | 0 | 0 | 0 | 0 | 0 | 0 |
|  | *Pachyteles mexicanus* | 2 | 0 | 0 | 6 | 0 | 0 | 0 | 0 | 0 |
|  | *Phloeoxena batesi* | 0 | 2 | 0 | 0 | 0 | 0 | 0 | 0 | 0 |
|  | *Platynus acutulus* | 0 | 1 | 0 | 0 | 0 | 0 | 0 | 0 | 0 |
|  | *Platynus amplicollis* | 0 | 0 | 0 | 0 | 1 | 0 | 0 | 0 | 0 |
|  | *Platynus cupripennis* | 0 | 0 | 0 | 0 | 2 | 0 | 0 | 0 | 0 |
|  | *Platynus variabilis* | 0 | 4 | 0 | 2 | 2 | 0 | 0 | 0 | 3 |
| **Dynastidae** | *Hemiphileurus dejeani* | 0 | 7 | 0 | 0 | 1 | 0 | 0 | 0 | 1 |
| **Leiodidae** | *Agathidium sp.* | 0 | 1 | 0 | 1 | 0 | 0 | 0 | 0 | 0 |
| **Passalidae** | *Heliscus tropicus* | 0 | 74 | 0 | 63 | 13 | 0 | 0 | 1 | 10 |
|  | *Odontotaenius striatopunctatus* | 0 | 0 | 0 | 0 | 0 | 0 | 0 | 0 | 1 |
|  | *Proculejus sp. (chica)* | 0 | 0 | 0 | 7 | 3 | 0 | 0 | 0 | 0 |
|  | *Proculejus sp. (grande)* | 0 | 0 | 0 | 0 | 1 | 0 | 0 | 0 | 0 |
|  | *Pseudacanthus aztecus* | 0 | 1 | 0 | 1 | 0 | 0 | 0 | 0 | 0 |
| **Ptilodactylidae** | *Ptilodactyla sp.* | 0 | 2 | 0 | 0 | 0 | 0 | 0 | 0 | 0 |
| **Scarabaeidae** | *Macraspis chrysis* | 0 | 0 | 0 | 0 | 0 | 0 | 0 | 0 | 1 |
|  | *Parisolea pallida* | 0 | 0 | 0 | 0 | 1 | 0 | 0 | 0 | 0 |
| **Staphyllinidae** | *Aleocharinae sp.2* | 0 | 0 | 0 | 0 | 4 | 0 | 0 | 0 | 0 |
|  | *Aleocharinae sp.3* | 0 | 0 | 0 | 1 | 0 | 0 | 0 | 0 | 0 |
|  | *Belonuchus sp.1* | 0 | 1 | 0 | 0 | 0 | 0 | 0 | 0 | 0 |
|  | *Bolitogyrus sp.1* | 0 | 0 | 0 | 0 | 0 | 0 | 0 | 0 | 1 |
|  | *Clavilispinus sp.1* | 0 | 0 | 0 | 2 | 2 | 0 | 0 | 0 | 4 |
|  | *Clavilispinus sp.2* | 0 | 0 | 0 | 0 | 1 | 0 | 0 | 0 | 0 |
|  | *Euconnus sp.* | 0 | 0 | 0 | 0 | 0 | 0 | 1 | 0 | 0 |
|  | *Homalolinus sp.1* | 0 | 4 | 0 | 1 | 4 | 0 | 0 | 0 | 2 |
|  | *Homalolinus sp.2* | 0 | 0 | 0 | 0 | 3 | 0 | 0 | 0 | 0 |
|  | *Leptochirus sp.1* | 0 | 0 | 0 | 1 | 8 | 0 | 0 | 0 | 1 |
|  | *Leptochirus sp.2* | 0 | 0 | 0 | 0 | 1 | 0 | 0 | 0 | 0 |
|  | *Misantlius sp.1* | 0 | 0 | 0 | 0 | 2 | 0 | 0 | 0 | 0 |
|  | *Neoxantholinus sp.1* | 0 | 0 | 0 | 0 | 1 | 0 | 0 | 0 | 0 |
|  | *Osorius sp.1* | 0 | 22 | 0 | 42 | 4 | 0 | 0 | 0 | 0 |
|  | *Priochirus sp.1* | 0 | 8 | 0 | 12 | 1 | 0 | 0 | 0 | 0 |
|  | *Priochirus sp.2* | 0 | 4 | 0 | 8 | 1 | 0 | 0 | 0 | 5 |
|  | *Sepedophilus sp.1* | 0 | 0 | 0 | 0 | 0 | 0 | 0 | 0 | 1 |
|  | *Suniocharis sp.1* | 0 | 0 | 0 | 1 | 2 | 0 | 0 | 0 | 0 |
|  | *Suniocharis sp.2* | 0 | 2 | 0 | 0 | 0 | 0 | 0 | 0 | 0 |
| **Tenebrionidae** | *Anaedus mexicanus* | 0 | 1 | 0 | 0 | 0 | 0 | 0 | 0 | 0 |
|  | *Arrhabaeus sp.* | 0 | 2 | 0 | 0 | 0 | 0 | 0 | 0 | 0 |
|  | *Diceroderes mexicanus* | 0 | 0 | 0 | 3 | 0 | 0 | 0 | 0 | 0 |
|  | *Platydema maculipennis* | 0 | 4 | 0 | 0 | 0 | 0 | 0 | 0 | 0 |
|  | *Uloma fossulata* | 0 | 1 | 0 | 0 | 0 | 0 | 0 | 0 | 0 |
|  | *Uloma mexicana* | 0 | 2 | 0 | 1 | 0 | 0 | 0 | 0 | 0 |
| **Zopheridae** | *Verodes asperatus* | 0 | 0 | 0 | 1 | 0 | 0 | 0 | 0 | 0 |
|  | **Total** | **2** | **145** | **0** | **153** | **62** | **0** | **1** | **1** | **31** |
